# Upregulation of glycolysis and oxidative phosphorylation in benzo[β]pyrene and arsenic-induced rat lung epithelial transformed cells

**DOI:** 10.18632/oncotarget.9814

**Published:** 2016-06-03

**Authors:** Huachen Chen, Lai-Sheung Lee, Guanwu Li, Sai-Wah Tsao, Jen-Fu Chiu

**Affiliations:** ^1^ Department of Biochemistry/Open Laboratory of Tumor Molecular Biology, Shantou University College of Medicine, Shantou, Guangdong, China; ^2^ School of Biomedical Sciences, LKS Faculty of Medicine, University of Hong Kong, Hong Kong, China

**Keywords:** arsenic, benzo[α]pyrene, Warburg effect, oxidative phosphorylation, hypoxia

## Abstract

Arsenic and benzo[β]pyrene (B[a]P) are common contaminants in developing countries. Many studies have investigated the consequences of arsenic and/or B[a]P-induced cellular transformation, including altered metabolism. In the present study, we show that, in addition to elevated glycolysis, B[a]P/arsenic-induced transformation also stimulates oxidative phosphorylation (OXPHOS). Proteomic data and immunoblot studies demonstrated that enzymatic activities, involved in both glycolysis and OXPHOS, are upregulated in the primary transformed rat lung epithelial cell (TLEC) culture, as well as in subcloned TLEC cell lines (TMCs), indicating that OXPHOS was active and still contributed to energy production. LEC expression, of the glycolytic enzyme phosphoglycerate mutase (PGAM) and the TCA cycle enzyme alpha-ketoglutarate dehydrogenase (OGDH), revealed an alternating cyclic pattern of glycolysis and OXPHOS during cell transformation. We also found that the expression levels of hypoxia-inducible factor-1β were consistent with the pattern of glycolysis during the course of transformation. Low doses of an ATP synthase inhibitor depleted endogenous ATP levels to a greater extent in TLECs, compared to parental LECs, indicating greater sensitivity of B[a]P/arsenic-transformed cells to ATP depletion. However, TLEC cells exhibited better survival under hypoxia, possibly due to further induction of anaerobic glycolysis. Collectively, our data indicate that B[a]P/arsenic-transformed cells can maintain energy production through upregulation of both glycolysis and OXPHOS. Selective inhibition of metabolic pathways may serve as a therapeutic option for cancer therapy.

## INTRODUCTION

Benzo[α]pyrene and arsenic derivatives are ubiquitously distributed in our environment [1–4], leading to wide-spread human exposure [5–10]. People living in contaminated areas have a high risk of getting various cancers [1, 2, 9, 11–14]. Various experimental models have been developed to understand how arsenic alone causes cancers [15–17], but these models are not comprehensive because arsenic alone is a weak carcinogen [17, 18] and cannot provide a complete understanding of the rising cancer incidence in areas chronically exposed to arsenic and B[a]P. Previously, our group revealed that the combined action of B[a]P and arsenic is 100-fold more tumorigenic than B[a]P or arsenic alone [18, 19]. Tumorigenicity of cells was confirmed by anchorage-independent growth and the ability to form tumors in nude mice. Using the SELDI-TOF Protein Chip, we were able to detect significant alterations of protein expression in transformed rat lung cells (TLECs), compared to parental LECs, with most of the altered proteins belonging to metabolic pathways [18]. Most cancer cells display up-regulation of glycolysis for fast production of ATP. This phenomenon was discovered 80 years ago and termed the “Warburg effect” [20]. A change in glucose metabolism has been implicated as a key contributor to malignant progression [21–23]. However, how this glycolytic switch happens, and whether it is a cause or a consequence has remained a matter of debate.

An increase in aerobic glycolysis does not mean that OXPHOS is deficient. Recent reports indicate that cancer cells preferring aerobic glycolysis show functional mitochondria, and that cancer cells can restore OXPHOS when glycolysis is inhibited [24–27]. Other reports also reveal that some cancers are more sensitive to inhibition of OXPHOS than inhibition of glycolysis [27, 28], indicating aerobic glycolysis is not a must for cancer cells. Due to the heterogeneity and plasticity of cancer cells, it is possible that such cancer cells rely more heavily on OXPHOS under normoxic conditions, and switch to glycolysis under hypoxic conditions.

## RESULTS

### B[a]P/Arsenic-induced lung epithelial cell transformation

**Figure 1:**
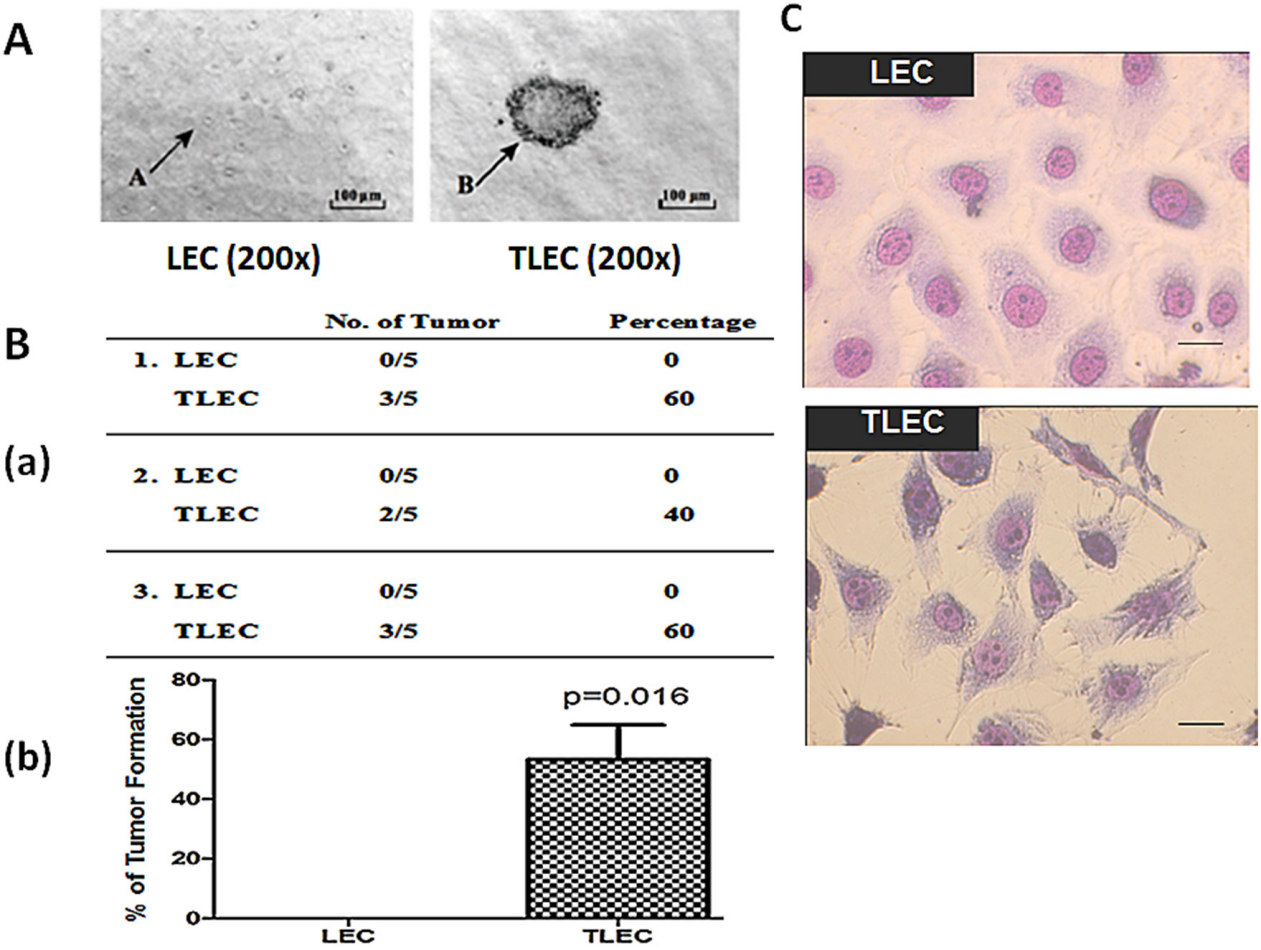
Morphology and tumorigenic properties of TLECs **A.** Soft-agar colony forming assay. Single cell suspensions of both LEC and TLEC cell lines were grown in semi-solid soft agar. Colony formation was examined under a microscope and photos were taken after incubation for 4 weeks. In the left graph, arrow A indicates a single LEC cell. In the right graph, arrow B points to a colony formed from TLEC cells. All images were taken at 200X magnification. **B.** Tumor formation in immunodeficient nude mice. a) Frequency of tumor formation in nude mice five weeks after subcutaneous injection with 5 × 10^6^ LEC or TLEC cells. b) Graphic representation of the percentage of nude mice developing tumors showing 60% and none of the mice developed tumors after subcutaneous injection with TLECs and LECs, respectively (*, p < 0.05 statistically significant difference compared with the control). **C.** Morphologies of LECs and TLECs. TLECs have larger nuclear size, and possess irregular cell size and shape, showing an aggressive and neoplastic phenotype. Bar = 10 μm.

Lung epithelial cell (LEC) transformation was induced by our previously-established transformation protocol [18, 19]. Transformation of LECs was evident by anchorage-independent growth in soft agar (Figure 1A). Transformed LECs (TLECs) gave an average of 20 colonies per well (seeded at 5 × 10^4^ cells per well) and the average diameter of the colonies was about 0.2 mm. In contrast, no colonies were observed upon inoculation of control LECs in soft agar. Morphologically, TLECs were characterized by a large nucleus, increased nucleus/cytoplasm ratio, and irregular size and shape, with movement starting with the formation of irregular cytoplasmic pseudopods (Figure 1C).

The tumorigenic properties of TLECs were studied *in vivo*. TLECs were injected into immunodeficient nude mice and examined for tumorigenicity. Five mice received subcutaneous injections of an equal number of cells (5 × 10^6^) of either control LECs or TLECs. Three repeat experiments were performed. In total, 8 out of 15 (53.3%) of mice injected with TLECs developed subcutaneous tumors at the site of injection, whereas none of the mice injected with control LECs developed tumors (Figure 1B). These data confirm the tumorigenic properties of TLECs *in vivo* and demonstrates that arsenic-induced cell transformation is a useful model for the characterization of events associated with the process of tumorigenesis.

### Up-regulation of the ATP synthase alpha-subunit

**Figure 2:**
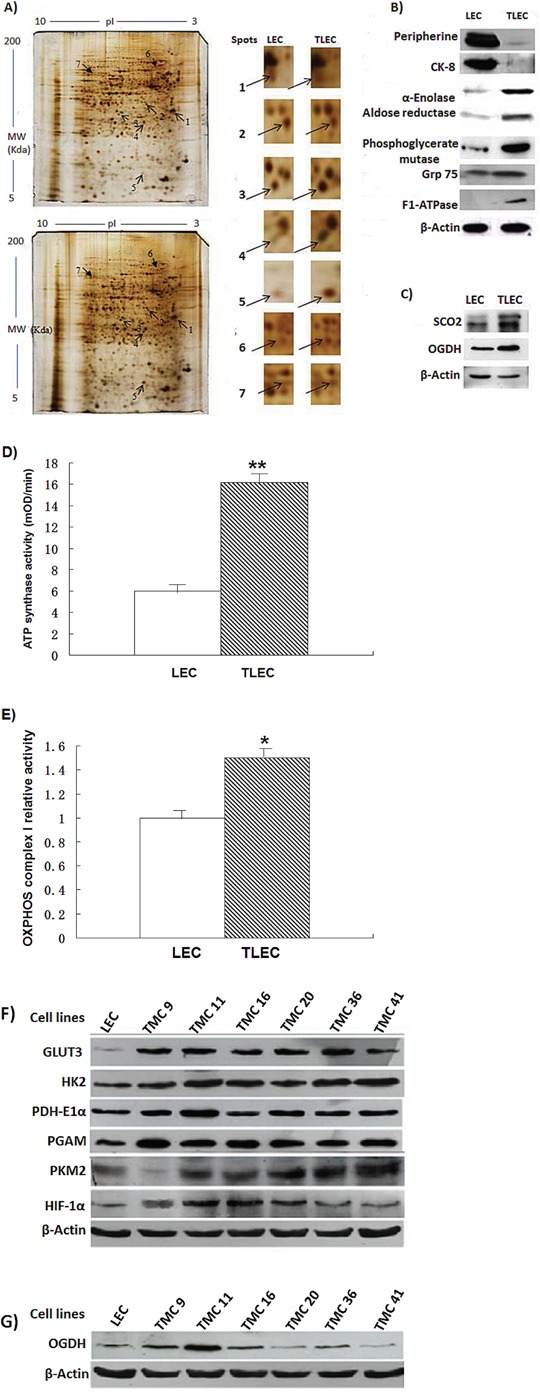
Representative protein gel images of parental LECs and transformed TLECs **A.** Protein pattern of control LECs (top) and TLECs (bottom) displayed by 2D gels (12.5%) and silver staining. Arrows identify the spots that were identified by MALDI-TOF mass spectrometry and listed in Table 1. **B.** Immunoblotting to validate the proteins identified by MALDI-TOF mass spectrometry. Total cellular proteins of LECs and TLECs were subjected to western blot analysis. After development, the membrane was stripped and re-probed with an actin monoclonal antibody to monitor the loading. **C.** Two enzymes in the OXPHOS pathway, SCO2 and OGDH, were determined by western blot analysis. Both SCO2 and OGDH were upregulated in TLECs. Results are representative of three independent experiments. **D.** and **E.** are enzymatic activities of ATP synthase and OXPHOS complex I in mitochondrial homogenates extracted from LECs or LECs, respectively. The amounts of protein from mitochondrial homogenate used for complex I was 20 μg and ATP synthase was 100 μg. Data are expressed as mean ± SD. *P*-value (Student's t-test) <0.05 was considered significant. **F.** and **G.** are immunoblots of glycolytic and OXPHOS enzymes in various transformed cell subclones (TMCs).

The protein profiles of parental LECs and TLECs were determined by tandem 2-dimensional electrophoresis and MALDI-TOF mass spectrometry. Several up-regulated and down-regulated proteins were detected in TLECs compared to LECs. Seven of these proteins, whose protein levels altered more than two-fold, are listed in Table 1. The proteomic data showed up-regulated proteins were involved in both glycolysis and chaperone functions (Figure 2A), validating the Warburg effect observed in arsenic exposed cells [34]. Apart from these glycolytic enzymes, up-regulation of the alpha-subunit of ATP synthase was also observed, suggesting that B[a]P/arsenic-transformed cells require greater ATP production due to alteration of enzymatic activities involved in both glycolysis and OXPHOS. The proteomic data was validated by immunoblotting (Figure 2B).

**Table 1:** Protein alterations in B[a]P/arsenic-transformed vs. parental cells

Spot No.	Protein	Accession No.	MW(Kd)/pI	Protein score/C.I.%	#protein matched/sequence	Fold difference ±S.D.
1	Peripherin	166063971	53/5.4	150/100	35/4	−10.94±0.34
2	Cytokeratin 8	40786432	52/5.4	134/100	32/5	− 2.54±0.97
3	Alpha-enolase	158186649	45/6.2	110/100	43/4	+ 2.55±0.01
4	Aldose reductase	6978491	36/5.9	183/100	43/20	+ 4.35±0.09
5	Phosphoglycerate mutase 1	16757984	29/6.7	261/100	48/4	+ 6.58±0.58
6	Grp75	10000439	74/5.87	100/100	16/2	+ 3.36±0.58
7	Chain A, rat liver	6729934	55/8.28	315/100	20/4	+ 6.40±1.14

Arsenic is a known inhibitor of OXPHOS. To show that OXPHOS is at least partly intact in TLECs, the expression of enzymes involved in OXPHOS was compared. SCO2 and OGDH are two important enzymes involved in OXPHOS. SCO2 is responsible for catalyzing the transfer of electrons from cytochrome c to oxygen and pump protons to generate the electrochemical gradient across the mitochondrial membrane, while OGDH is crucial for catalyzing the conversion of alpha-ketoglutarate to succinyl-CoA, an intermediate substrate in the tricarboxylic acid cycle. These two enzymes were up-regulated in TLECs (Figure 2C), suggesting that OXPHOS was active.

To ensure the increase in protein levels of ATP synthase and enzymes involved in OXPHOS reflected an increase in their enzymatic activities, we next quantified checked the activities of the individual proteins. In this experiment, we used an ATP synthase activity kit which first captures the ATP synthase complex in the reaction wells and then measures the activity by the oxidation of NADH to NAD^+^. Our measurements show that ATP synthase activity was enhanced 2.7-fold in TLEC cells (Figure 2D). OXPHOS complex I enzyme activity was increased by 76% in TLEC cell extracts (Figure 2E).

TLECs are heterogeneous transformed cells and each transformed cell may have its individual metabolic features. We therefore subcloned TLECs into several transformed cell lines designated as TMC1, TMC2, and so on. As shown in Figure 2F, their glucose metabolic phenotypes varied from one TMC to another. Although aerobic glycolysis was favored in TLECs, OXPHOS still contributed to energy production in some TMCs, and may play a significant role in their energy production (Figure 2G).

### TLECs are susceptible to both inhibition of OXPHOS and glycolysis

Our proteomic data revealed that enzymes involved in both glycolysis and ATP synthase were up-regulated in TLECs, suggesting that both glycolysis and OXPHOS were stimulated. To determine if TLECs were susceptible to both inhibition of glycolysis and OXPHOS, cellular viability was examined after addition of a glycolysis inhibitor or OXPHOS inhibitor. 2-Deoxy-D-glucose (2DG) is an analog of glucose and able to inhibit glycolysis [35]. Sodium azide inhibits cytochrome oxidase, which is the complex IV in the electron transport chain, and therefore inhibits ATP production from mitochondria [36].

**Figure 3:**
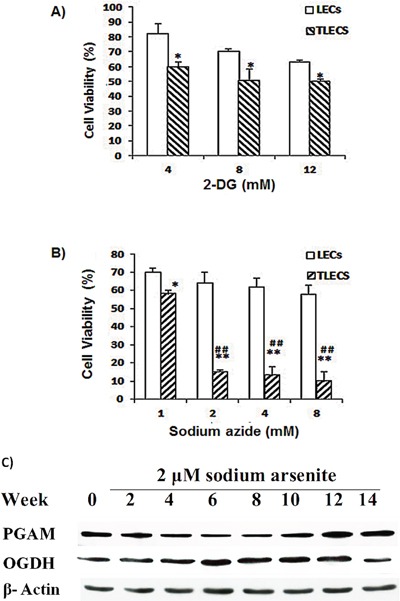
Effects of 2-DG and sodium azide on cell viability of LECs and TLECs 2DG is an inhibitor of glycolysis and sodium azide is an inhibitor of OXPHOS. Cell viability decreased as increasing concentrations of **A.** 2DG or **B.** sodium azide were added to cell culture medium for 24 h. TLECs showed significant cell death at concentrations of sodium azide higher than 2 mM. Cell viability was determined by NBB staining as described in the Materials and Methods section (*, p < 0.05 compared to LECs at the same concentration; **, p < 0.001 compared to LECs at the same concentration; ##, p < 0.001 TLECs at concentration of 1 mM sodium azide). **C.** Immunoblots of PGAM and OGDH enzymes in various transformed cell subclones.

Cytotoxicity was examined in response to increasing concentrations of 2DG. A dose-dependent cytotoxicity was observed in both LECs and TLECs (Figure 3A). Consistent with the literature, arsenic-transformed TLECs were more susceptible to inhibition of glucose metabolism by 2DG [34], suggesting that TLECs rely more on glucose metabolism than parental LECs. To determine cytotoxicity towards inhibition of OXPHOS, increasing concentrations of sodium azide were added to cells. At concentrations above 1 mM, TLECs exhibited significantly more cell death compared to LECs (Figure 3B). Together, our findings suggest TLECs rely on both glycolysis and ATP production from mitochondria.

By comparing the glycolytic enzyme PGAM and TCA cycle enzyme OGDH, we also found interesting cyclic patterns of glycolysis and OXPHOS in LECs during the course of cell transformation. As shown in Figure 3C, glycolytic enzymes increased at an early stage of transformation, decreased in the midterm, and then increased again at a later stage during transformation process. The pattern of OXPHOS was the reverse, with OGDH levels decreasing initially, then increasing significantly during the mid-term of transformation, and finally decreasing at the latter stage of progression. This phenomenon is consistent with Smolkova et al. [37].

### ATP synthase plays an important role in the metabolism of TLECs

**Figure 4:**
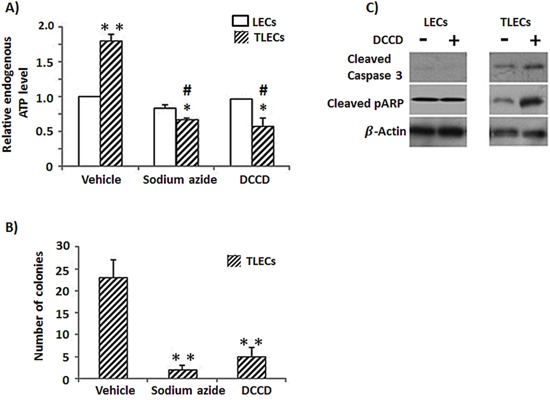
Reduction of cellular ATP content induces expression of apoptotic proteins and reduces tumorigenicity in TLECs **A.** Endogenous ATP content was determined by an ATP kit as described in the Materials and Methods. TLECs showed elevated endogenous ATP levels compared to parental LECs. DCCD is an inhibitor of ATP synthase and can inhibit ATP production from oxidative phosphorylation. Application of a low dose of sodium azide (5 mM, 24 h) and DCCD (50 μM, 24 h) independently reduced endogenous ATP levels of TLECs, but parental LECs showed negligible change. **B.** TLECs were treated with sodium azide (5 mM) or DCCD (50 μM) for 24 h. Cells were collected and allowed to grow in soft agar for four weeks. Only colonies with a diameter larger than 60 μm were counted. The number of colonies was determined from an average of 8 random fields. Inhibition of ATP production reduced the tumorigenicity of TLECs, as indicated by reduced ability for anchorage-independent growth. (*, p < 0.05 compared to LECs at the same concentration of either sodium azide or DCCP; **, p < 0.001 compared to LECs; #, p < 0.05 compare to TLECs when only vehicle was added.) **C.** Protein lysate after DCCD (50 μM, 24 h) treatment was subjected to western blot analysis. Proteins were probed for apoptotic markers cleaved caspase 3 and cleaved PARP. Membranes were stripped and re-probed with actin antibody to monitor loading differences. Inhibition of ATP production induced expression of cleaved caspase 3 and cleaved PARP in TLECs, but not in parental LECs.

Our proteomic data and enzymatic activities showed an induction of the alpha-subunit of ATP synthase in TLECs, and our cytotoxicity test suggested that ATP production from mitochondria is important in TLECs. We then compared endogenous ATP levels in both LECs and TLECs. Consistent with our proteomic findings, a higher endogenous ATP level was found in TLECs compared to parental LECs (Figure 4A). To determine the change in endogenous ATP level after inhibition of ATP production by mitochondria, sodium azide and N,N′-dicyclohexylcarbodiimide (DCCD) were applied. DCCD is a specific inhibitor of ATP synthase, and inhibits ATP synthase by blocking the proton flux [38]. Both sodium azide and DCCD were used at low concentrations that caused negligible changes in the endogenous ATP level in parental LECs. However, the same dose of sodium azide and DCCD resulted in a nearly 30% reduction of endogenous ATP levels in TLECs (Figure 4A). Our data indicate that ATP synthase is important for ATP production in B[a]P/arsenic-transformed TLECs. Reduced ATP levels dramatically lowered the ability of TLECs to grow independently in soft agar (Figure 4B), indicating inhibition of ATP synthesis was able to inhibit the *in-vitro* transforming ability of TLECs. Higher expression of apoptotic proteins, such as cleaved caspase 3 and cleaved PARP, were found in TLECs in response to DCCD treatment, but not in parental LECs (Figure 4C), suggesting that reductions in ATP level in TLECs lead to apoptosis.

### Ability to switch to glycolysis under hypoxia

**Figure 5:**
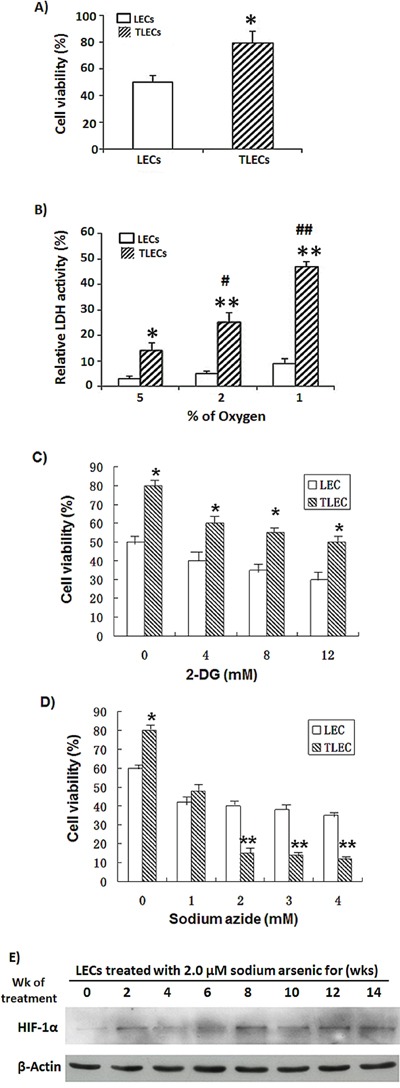
Effects of hypoxia on glycolysis and viability of TLECs **A.** LEC and TLEC cells were placed in a hypoxic incubator (under 2% oxygen) for 24 h. Cell viability was determined by NBB staining. TLECs were more adapted to hypoxic environment and showed higher cell viability than LECs. **B.** Both LEC and TLEC cells were incubated in various oxygen concentrations for 24 h, respectively, and glycolytic activity was determined by LDH assay. LDH activity is reflective of the level of anaerobic respiration. TLECs showed a higher LDH surge compared to parental LECs, indicating TLECs could rapidly induce glycolysis under hypoxia (*, p < 0.05 compared to LECs; **, p < 0.001 compared to LECs at the same oxygen percentage; #, p < 0.05 compared to TLECs when the oxygen percentage was set at 5%; ##, p < 0.001 compared to TLECs when the oxygen percentage was set at 5%). Both LEC and TLEC Cells were put in a hypoxic incubator (under 2% oxygen) for 24 h. Cell viability decreased as increasing concentrations of **C.** 2DG or **D.** sodium azide were added to cell culture medium for 24 h. TLECs showed signification cell death at concentrations of sodium azide higher than 2 mM. Cell viability was determined by NBB staining as described in the Materials and Methods section (*, p < 0.05 compared to LECs at the same concentration; **, p < 0.001 compared to LECs at the same concentration). **E.** Immunoblotting of HIF-1α in LEC cells at various time points during the transformation process. Total cellular proteins of LECs at various time points were subjected to western blot analysis with HIF-1α antibody. After development, the membrane was stripped and re-probed with an actin monoclonal antibody to monitor the loading difference.

Recently we reported that TLECs display a high level of reactive oxygen species (ROS) compared to LEC cells [39]. Here, we also show an increase of antioxidants and hypoxia inducible factor-1α (HIF-1α) in TLECs (Figure 2D). It has been demonstrated that increasing the level of ROS could, through HIF-1, regulate gene expression involved in glycolysis in response to hypoxia [40]. Under hypoxic conditions, oxygen supply is limited and OXPHOS is hampered. Most cancer cells will rely more on glycolysis for energy production under hypoxia. Our findings showed that TLECs rely on both OXPHOS and glycolysis under normoxia. To examine if TLECs could survive better under hypoxic conditions, cells were cultured under 2% oxygen and cellular viability was determined. Our data, shown in Figure 5A, indicate that the viability of TLECs was higher (80%) as compared to LECs (50%) under 1% oxygen, suggesting that TLECs are more adaptable to hypoxic conditions and can maintain energy production to support their growth. To determine if TLECs could switch to anaerobic glycolysis under hypoxia, LDH activity was measured. LDH is the enzyme responsible for the conversion of pyruvate to lactate under anaerobic glycolysis [33]. LDH activity can therefore reflect the degree of anaerobic glycolysis. Parental LECs only showed a small increase of LDH activity, whereas a surge of LDH activity was observed in TLECs under decreasing concentrations of oxygen content (Figure 5B), indicating that TLECs were able to stimulate anaerobic glycolysis rapidly under hypoxia.

To further determine which of two energy production pathways, glycolysis and OXPHOS, LECs and TLECs rely on more during hypoxic conditions, cellular viability was examined after addition various amounts of the glycolysis inhibitor 2DG or OXPHOS inhibitor sodium azide to media of cells cultured under 2% oxygen. As shown in Figure 5C and 5D, TLECs showed significant cell death as compared to LEC when cells treated with sodium azide concentrations higher than 2 mM.

Next we examined the expression levels of HIF-1α in LECs during the course of transformation process induced by B[a]P/arsenic. As shown in Figure 5E, the expression level of HIF-1α increased initially, then decreased at the mid-stage of transformation. The level of HIF-1α started to increase again at 10th-week of treatment and continued to stay at a high level.

## DISCUSSION

### Induced glycolysis and OXPHOS were observed in B[a]P/arsenic-transformed cells

Chronic B[a]P and arsenic co-exposure is still common in developing countries [5, 6, 11]. It has been known for many years that chronic arsenic exposure is associated with a rising incidence of cancer [10]. However, the underlying mechanisms are still unclear. Our previous data showed that combined action of arsenic and B[a]P is 100-fold more tumorigenic than B[a]P or arsenic alone [18, 19]. Thus, studies examining arsenic alone are not comprehensive and may not provide a full explanation for the observed effect of arsenic. Here we have established a TLEC model in which B[a]P-initiated LECs are made tumorigenic by chronic exposure to low doses of sodium arsenite [18, 19]. This cell model is closer to the real situation found in developing countries.

This study, as well as our previous results [18], shows that glucose metabolism is up-regulated in TLECs, as is evident by induced expression of glycolytic enzymes (Figure 2B). Consistent with this finding, a later report also found that arsenic exposure can induce the Warburg effect in cultured cells [34]. TLECs exhibit profound bioenergetic and histological differences as compared to their non-transformed counterpart. All these modifications are associated with increasing of cell growth, inhibition of apoptosis and intense anabolism.

**Figure 6:**
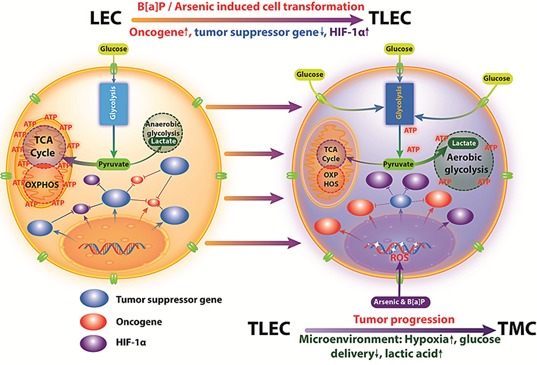
TLECs show interplay between glycolysis and OXPHOS to produce ATP In LECs, OXPHOS is the major source for ATP production. However, TLECs shifted from OXPHOS to glycolysis for the production of energy (Warburg effect). The increased level of ROS production in TLECs has been shown to inhibit prolyl hydroxylase enzyme (PHD) activity, thus preventing HIF-1α proteasomal degradation. Consequently, HIF-1α is stabilized and translocates to the nucleus, where it dimerizes with HIF-1β, initiating the transcription of HIF-1 responsiveness genes including most of the glycolytic enzymes that promote glycolysis in TLECs. However, in response to different microenvironments, hypoxia conditions, and the sequence of oncogenic activation and tumor suppressor gene inactivation, TLECs may express upregulation of OXPHOS components.

The Warburg effect was discovered almost a century ago and in this hypothesis, cancer cells prefer to produce energy by upregulating glycolysis rather than OXPHOS, even when the oxygen supply is not limited [20]. Warburg proposed that aerobic glycolysis in cancer cells is due to a permanent impairment of mitochondrial OXPHOS [41]. Recently, however, many investigators found that the function of mitochondrial OXPHOS in most cancers is intact [42–47], and OXPHOS is preferentially stimulated in some cancer cells [27, 28]. Aerobic glycolysis in many cancers may be caused by various (Figure 6). Several investigators also have suggested that the Warburg effect in cancer is due to enhanced glycolysis suppressing OXPHOS rather than defects in mitochondrial OXPHOS. If glycolysis is suppressed in cancer cells, the function of mitochondrial OXPHOS should be able to be restored [28, 37, 44, 48]. Reitzer and colleagues [49] have demonstrated that OXPHOS is used preferentially to produce ATP in cervical carcinoma cells. Griguer et al. further identified two types of glioma cell lines, i.e. glycolytic and OXPHOS gliomas [43]. This flexibility in the interplay between glycolysis and OXPHOS in cancer cells is required by the mechanisms of energy production to respond to microenvironmental changes, as well as differences in tumor energy needs or biosynthetic activity. The present study also shows that within TLEC tumors are different transformed cells with various metabolic phenotypes (Figure 2F and 2G), indicating different subclones of transformed cells within TLEC benefit each other in metabolism and form a metabolic symbiont. Some cloned transformed LECs (TMC cells) demonstrate a reduction in oxidative phosphorylation (OXPHOS) capacity, whereas other TMCs express up-regulation of OXPHOS components (Figure 2F and 2G). This difference in bioenergetic types of TMCs can be explained by differences in microenvironment, hypoxia conditions, and the sequence of oncogenic activation and tumor suppressor gene inactivation during carcinogenesis and tumor progression (Figure 6). This switch is also observed at the level of glutamine oxidation, as shown by increasing OGDH enzyme activity (Figure 2E and Figure 3C). In sum, we might consider that OXPHOS and glycolysis cooperate to sustain energy needs during tumorigenesis, as clearly shown in Figure 3C. Recently Smolkova and colleagues also demonstrated waves of suppression and then restoration of oxidative phosphorylation in cancer cells [37].

It is now known that cancer cell preferences for glycolysis are due to the rapid production of energy [41] rather than going through OXPHOS. However, that the question arises as to whether up-regulation of glycolysis is the best way for cancer cells to produce energy. In the present study, we show that both glycolysis and OXPHOS are up-regulated in TLECs (Figures 2C, 2D and 2E). This finding suggests that these two metabolic pathways are not mutually exclusive. We hypothesize that stimulation of both glycolysis and OXPHOS will provide more energy in a shorter time to meet the high energy demand for cancer cells, which is especially important for those cancers that have intrinsic defects in energy production.

### OXPHOS is at least partially intact in B[a]P/arsenic-transformed cells

Arsenic is known to inhibit OXPHOS. Arsenic can inhibit pyruvate dehydrogenase [50, 51], which converts pyruvate to acetyl-CoA, the substrate of OXPHOS. Arsenic can also inhibit succinic dehydrogenase [52], the complex II of the electron transport chain. Arsenic is a phosphate analog [53] that can uncouple OXPHOS and reduce energy production. On the contrary, cancer cells require high energy production to support their rapid growth and expansion. Arsenic-exposed cells need to find a way to overcome an intrinsic energy deficient problem before undergoing transformation. The initial clue we had, from a previous independent proteomic study, was that stimulation of glycolysis compensates for reduced OXPHOS [34]. Our proteomic study and enzymatic activity assays show that enzymes involved in OXPHOS are also up-regulated in TLECs (Figure 2C and 2E). To validate that OXPHOS is at least partially intact in TLECs, two additional enzymes, SCO2 and OGDH (Figure 2F and 2G), involved in the process were also studied. In sum, our data suggests that OXPHOS is functioning in TLECs. We hypothesize that chronic exposure to low dose arsenic does not completely inhibit OXPHOS, but may hamper the process. Up-regulation of ATP synthase by TLECs may compensate for the energy loss induced by arsenic (Figure 2B and 2D).

### B[a]P/arsenic-transformed cells are able to further stimulate glycolysis in hypoxia

Energy production in a hypoxic environment relies mainly on glycolysis, rather than OXPHOS, due to the limited supply of oxygen [23, 54–57]. HIFs induce transcription factors to expedite cellular adaptation to hypoxic environments and play critical roles in shifting from the OXPHOS to the glycolytic phenotype in cancer [58–61]. HIF-1α regulates more than one hundred different genes, including GLUT1 and GLUT3, and up-regulates nine of the ten glycolytic enzymes [62]. It also activates pyruvate dehydrogenase kinase 1 (PDK1), which inhibits the conversion of pyruvate to acetyl CoA and therefore decreases mitochondrial OXPHOS. Significantly, a recent study demonstrated that pyruvate kinase M2 (PKM2) gene transcription is also activated by HIF-1α [63]. PKM2 is critical for aerobic glycolysis and tumor growth [64]. M type pyruvate kinase (PK) has two isoforms: PKM1 and PKM2. PKM1 is expressed in most adult tissue, whereas PKM2 is only expressed in embryonic and proliferating tissues.

Previously, our laboratory demonstrated an increased level of ROS during LEC transformation induced by B[a]P/arsenic [29, 65]. ROS subsequently induced HIF-1α. HIF-1α, in turn, stimulated the expression of glycolytic enzymes and glucose transporters Glut 1 and Glut 3. In the present study, we found that TLECs could undergo a further stimulation of glycolysis, in a hypoxic environment, compared to parental LECs, indicating TLECs are versatile in selecting metabolic processes to meet their energy demand and maintain growth. When TLECs are located in well-oxygenated conditions, they express aerobic glycolysis. However, when TLECs are present under hypoxic conditions, hypoxia promotes “anaerobic glycolysis” through HIF-1α-dependent up-regulation. Cells using aerobic or anaerobic glycolysis survive only in case they extrude acidic metabolites acidifying the extracellular space. Acidosis drives TLECs from glycolysis to OXPHOS. Therefore, TLECs are more resistant to the glycolysis inhibitor 2-DG and more sensitive to the OXPHOS inhibitor sodium azide (Figures 3A and 3B, Figures 5C and 5D). The ability of OXPHOS inhibition (e.g. sodium azide) to preferentially kill transformed cells (TLECs) in a hypoxic environment provides a biochemical basis to further develop this class of compounds as novel anticancer therapeutic agents.

In conclusion, the present study expands our understanding of energy producing pathways in B[a]P/arsenic-transformed TLEC cells. In addition to stimulation of glycolysis, TLECs can also up-regulate OXPHOS to enhance energy production for growth and expansion during carcinogenesis. Our study demonstrates that TLECs can rapidly stimulate further induction of glycolysis under hypoxia, providing a growth advantage in a hostile environment. Alteration of energy metabolism in cancer cells may be a therapeutic target of choice. Understanding the features and complexity of the cancer energy metabolic profile may help to develop new approaches in early diagnosis and effectively target therapy of cancer.

## EXPERIMENTAL PROCEDURES

### Cell culture

Rat lung epithelial cells (LECs) [29] were cultured in F-12 medium (GIBCO-BRL, Grand Island, NY) supplemented with 1.6 g/L sodium bicarbonate (Sigma, St. Louis, MO), 10% (v/v) fetal bovine serum (FBS, GIBCO, Invitrogen, Carlsbad, CA) and 100 U/ml penicillin- streptomycin (GIBCO-BRL). Cultures were maintained in a humidified incubator with 95% air and 5% CO_2_ at 37°C. For hypoxia experiments, cells were cultured under 1%, 2%, or 5% oxygen for various times at 37°C. 2-Deoxy-D-glucose (2DG, Sigma, St. Louis, MO) and sodium azide (NaN3, Sigma) were dissolved in Milli-Q water and filtered through a 0.22 μM membrane for sterilization. N,N′-dicyclohexylcarbodiimide (DCCD, Sigma) was dissolved in DMSO and filtered through a 0.22 μM membrane. Designated concentrations of each inhibitor were added to cells for the indicated times.

### Arsenite-induced *in vitro* cell transformation

Transformation of LECs was performed as described in our previous paper [18]. Briefly, LECs were cultured in medium with 100 nM benzo[α]pyrene (Sigma, St. Louis, MO) for 24 h. Then medium was replaced with medium containing 2 μM sodium arsenite (Sigma). These LECs were allowed to grow in the arsenite-containing medium for 12 weeks. Cell transformation was determined by capacity for anchorage-independent growth and *in-vivo* tumor formation in nude mice. For tumor formation in nude mice, 5 × 10^6^ LECs or TLECs were inoculated subcutaneously into 3- to 4-week-old female Nu/Nu nude mice. Tumor formation was assessed every three days for five weeks. This nude mouse tumorigenicity assay was repeated three times with 5 mice in each group.

### Western blotting

Western blot analysis was performed as described [30]. Fifteen μg total protein lysate was separated on 10% SDS-PAGE. Proteins were then transferred to a PVDF membrane (Amersham Biosciences, Piscataway, NJ). The membrane was blocked, incubated with primary antibodies overnight and secondary antibody for 1 h at room temperature. The membrane was developed with ECL Plus chemiluminescence reagent (Amersham Biosciences). The primary antibodies used in this study were against actin (Santa Cruz, CA), peripherin (Santa Cruz, CA), cytokeratin-8 (Abcam, MA), aldose reductase (Abcam, MA), alpha-enolase (Santa Cruz, CA), Grp75 (Cell Signaling Technology, MA), phosphoglycerate mutase (Santa Cruz, CA), F1 ATPase (Santa Cruz, CA), SCO2 (Santa Cruz, CA) and OGDH (Santa Cruz, CA).

### Proteomic study

Two-dimensional electrophoresis, tryptic in-gel digestion and MALDI-TOF-MS analysis were conducted by following a protocol described previously [31]. Briefly, 300 μg whole cellular lysate was diluted in rehydration solutions containing a trace amount of bromophenol blue. Isoelectric focusing (IEF) was carried out with precast 13 cm ImmobilineDryStrips (IPG strips, Amersham Biosciences) to generate a non-linear pH gradient of 3-10. Following IEF, strips were immediately used for the second dimension SDS-PAGE. Proteins were visualized with silver stain, and two-dimensional gels were scanned by using an Image Scanner (Amersham Biosciences), operated by LabScan 3.00 software. Intensity calibration was performed with an intensity step wedge prior to gel image capture. Image analysis was carried out by ImageMaster 2D Elite software 4.01. Only up-/down-regulated spots (± over 1.5-fold) or spots that either appeared or disappeared were selected for tryptic in-gel digestion followed by analysis with a mass spectrometer (MS).

### Cell viability assay

Cell viability was measured by a naphthol blue black (NBB) staining assay [32]. Cells were plated in 96-well plates at a density of 2 × 10^4^ per well. Cells were treated under various conditions at the indicated times. At the end of the experiment, cells were fixed in 10% formalin for 10 min and stained with NBB solution (0.05% NBB in 9% acetic acid with 0.1 M sodium acetate) for 30 min at room temperature. Fixed cells were washed three times with water to remove free dye. The attached dye was eluted with 150 μl of 50 mM NaOH. The optical density at 595 nm was measured using a Model EI 310 Autoplate reader (Bio-Tek Instrument, Winooski, VT).

### Enzyme activity assays

The enzymatic activity of ATP synthase, which is responsible for ATP generation, was also assessed in the LECs and TLECs using an ATP Synthase Enzyme Activity Microplate Assay Kit (ab109714, Abcam, Cambridge, MA, USA) according to the manufacturer's protocol. Briefly, ATP synthase from each cell culture was selectively immunocaptured in the microplate wells, and the ability of the captured ATP synthetase to synthesize ATP was determined by detecting the change in absorbance at 340 nm using a microplate reader (Synergy HT, BioTek, VT). These activities were expressed as a change in absorbance per minute per milligram of protein.

Complex I activities were analyzed using the Complex I Enzyme Activity Microplate Assay kit (ab109721, Abcam, Cambridge, MA). The complex I activity was measured by following the oxidation of NADH to NAD^+^ and the simultaneous reduction of a dye which leads to increased absorbance at 450 nm.

### Measurement of cellular ATP content

ATP concentration was determined by an ATP kit according to manufacturer's protocol (PerkinElmer, Boston, MA). Briefly, cells were cultured in 96-well plates at density 5 × 10^3^ cells/well. ATP inhibitors were added to the wells. At the end of the incubation period, 50 μl of mammalian cell lysis solution provided in the kit was added to the culture medium and the plate was shaken for 5 min in the dark. Fifty μl substrate solution was added into each well. The plate was shaken for 5 min in the dark, and then luminescence was measured by a plate reader.

### Measurement of glycolytic activity

Cellular glycolytic activity was determined by measuring the activity of the key enzyme, lactate dehydrogenase (LDH). The activity of lactate dehydrogenase was monitored spectrophotometrically by the increase in NADH at 340 nm when pyruvate was converted to lactate [33]. Lactate concentration in the culture medium was measured using commercial chromatometric kits from Sigma (St. Louis, MO).

### Statistical analysis

Statistical analysis was performed by using a two-tailed Student's t-test, and a p < 0.05 was considered significant. Data are expressed as the mean ± SD of triplicate samples, and the reproducibility was confirmed in three separate experiments.

## Abbreviations

B[a]P: benzo[α]pyrene
DCCD: N,N′-dicyclohexylcarbodiimide
2DG: 2-deoxy-D-glucose
IEF: isoelectric focusing
LDH: lactate dehydrogenase
LEC: lung epithelial cell
NBB: naphthol blue black
SCO2: synthesis of cytochrome c oxidase 2
OGDH: alpha-ketoglutarate dehydrogenase
OXPHOS: Oxidative phosphorylation
TLEC: transformed lung epithelial cell
